# Controlled Synthesis of Visible Light Active Cu_x_S Photocatalyst: The Effect of Heat Treatment on Their Adsorption Capacity and Photoactivity

**DOI:** 10.3390/ma13173665

**Published:** 2020-08-19

**Authors:** Szilvia Fodor, Lucian Baia, Klára Hernádi, Zsolt Pap

**Affiliations:** 1 Department of Applied and Environmental Chemistry, University of Szeged, Rerrich sqr. 1, HU-6720 Szeged, Hungary; fod_szilvia@chem.u-szeged.hu; 2 Nanostructured Materials and Bio-Nano-Interfaces Centre, Institute for Interdisciplinary Research on Bio-Nano-Sciences, Treboniu Laurian str. 42, RO–400271 Cluj-Napoca, Romania; lucian.baia@phys.ubbcluj.ro; 3 Faculty of Physics, Babeș–Bolyai University, M. Kogălniceanu 1, RO–400084 Cluj–Napoca, Romania; 4 Institute of Environmental Science and Technology, Tisza Lajos blvd. 103, HU-6720 Szeged, Hungary; 5 Institute of Research-Development-Innovation in Applied Natural Sciences, Babes-Bolyai University, Fântânele str. 30, 400294 Cluj-Napoca, Romania

**Keywords:** copper sulfide, visible light, photocatalysis

## Abstract

The effects of different precursor salts, stabilizing agents, and heat treatment parameters are already known to have an influence on the synthesis of nano-sized semiconductors in heterogenous photocatalysis. In the present work, CuxS materials were prepared by using different precursors (copper (II) chloride dihydrate or copper (II) acetate monohydrate) and shape tailoring/stabilizing agents, such as ethylenediaminetetraacetic acid/polyvinylpyrrolidone, and thiourea as the sulfur source. The polyvinylpyrrolidone (PVP) kinetically controlled the growth rate of the nanoplates, while ethylenediaminetetraacetic acid (EDTA) adjusted the nucleation process through the complexation of copper. A one-step hydrothermal method was used for the synthesis, and the materials were characterized by means of morphological and structural complementary investigation methods. Furthermore, the adsorption capacity and photocatalytic activity were also measured for these materials. It was found that the vacancy sites formed by changing the precursor salt, as confirmed by Raman measurements, affect the photocatalytic activity. The rise of the specific surface area was achieved by heat treatment, and concomitantly, the adsorption capacity of the treated samples was found to increase likewise.

## 1. Introduction

Metal sulfides have been paid much attention and were investigated recently because of the variability in their structure and potential applications, such as in solar cells [1,2], gas sensors [3], and plasmonic applications [4,5]. Their applicability includes photocatalytic water treatment due to the broad light absorption range and promising photocatalytic performance [6,7,8] of metal sulfide-based semiconductors (CuS, ZnS, CdS).

Cu_x_S is a well-known p-type semiconductor, which can be synthesized by relatively simple ways and has a relatively low production cost. Cu_x_S can be found as stable chalcolite (Cu_2_S) or covellite (CuS), and in the form of certain stable and metastable phases with varying stoichiometries between Cu_2_S and CuS [9]. The latter ones are characterized by high stability, low toxicity, and high photothermal conversion efficiency [10]. The bandgap energy of Cu_x_S materials is between 1.2 eV and 2.5 eV, clearly pointing out visible light excitability [11,12,13].

Depending on the synthesis method and experimental parameters, Cu_x_S forms different phases with a varying stoichiometry between Cu and S [14]. Therefore, in general, the crystalline phase composition and the resultant optical properties can be tuned by choosing selective synthesis methods of Cu_x_S.

The choice of the synthesis parameters and conditions can greatly influence the structural and morphological properties of the materials, while directly influencing their applicability. One possible starting point is the choice of the precursor salt, which plays an important role in the development of different morphological features [15]. This may be due to the fact that different anions have different affinities toward Cu^2+^ [16], and they also interact differently with the surface of the product [17].

Stabilizer agents may have a similar effect on the formation of particles: sodium dodecyl sulfate (SDS) [18], ethylenediaminetetraacetic acid (EDTA) [19], or polyvinylpyrrolidone (PVP) [20] are known to be applied successfully. Polymer stabilization can be also applied in the synthesis of metal sulfides, the most frequent one being the already mentioned PVP. The advantages of preparing the polymer-stabilized particles is the ability to control particle size, enhance their stability, and maintain consistent morphology [21].

The major role of EDTA in the synthesis procedure of Cu_x_S can be explained by the fact that EDTA and Cu^2+^ can form a complex compound (a chelate), which can prevent the total reduction of Cu^2+^ to Cu, conferring a higher flexibility to the synthesis procedure [22]. It should be noted that the reduction procedure may depend on the available sulfur source as well. In the synthesis of Cu_x_S ammonium sulfide [23], sodium thiosulfate [24] and thiourea [25,26] were already applied. The latter one can be used as a reducing agent as well [27,28].

Another influencing factor for these materials may be an additional heat treatment. The term of calcination is used for the classical heat treatment procedure in most of the publications, but sometimes, it is used for solvothermal crystallization [29] as well. After heat treatment, structural changes can be observed in metal sulfide samples [30]: heating in air/oxygen (oxidative atmosphere) causes the stoichiometry of Cu_x_S to change, including the appearance of sulfates, while heating in hydrogen (reductive) atmosphere results in the appearance of reduced species, such as metallic Cu. All the mentioned alterations may also affect photocatalytic activity. Therefore, the use of an inert atmosphere may be justified to avoid the formation of Cu_x_O by chemical oxidation.

Therefore, to clarify the above-mentioned issues, the structure, the surface area, and the photocatalytic activity of the Cu_x_S and Cu_x_S_C calcined samples were investigated in the current study using different precursor salts and stabilizing agents.

## 2. Materials and Methods

### 2.1. Materials

In a typical synthesis process, copper (II) chloride dihydrate (CuCl_2_ 2H_2_O, Alfa Aesar, Karlsruhe, Germany, 99+%) or copper (II) acetate monohydrate (Cu(Ac)_2_–Cu(OOCCH_3_)_2_ H_2_O, Alfa Aesar, Karlsruhe, Germany, 98+%) was used as precursor dissolved in ethanol (VWR, Fontenay-sous-Bois, France, 98%). As a stabilization agent, ethylenediaminetetraacetic acid (EDTA, C_10_H_16_N_2_O_8_, Molar, Halásztelek, Hungary, 99.5%) or polyvinylpyrrolidone ((C_6_H_9_NO)_n_, Sigma-Aldrich, St Louis, MO, USA, Mw ≈ 40,000) was used. Additionally, thiourea (CH_4_N_2_S, Molar, 99.98%) was added as a sulfur source. For the purification step, ultrapure Milli-Q water and ethanol were used. All the chemicals were purchased and used without further purification.

### 2.2. Synthesis of Cu_2_S Microparticles

Cu_2_S was obtained via one-step hydrothermal method in a Teflon^®^-lined autoclave. In a typical synthesis, 0.5 g of copper (II) chloride (CuCl_2_ 2H_2_O), 0.4 g thiourea (CH_4_N_2_S), and a proper amount of stabilization agent (1.5 g ethylenediaminetetraacetic acid) was dissolved in 40 mL of ethanol. The solution was kept under vigorous stirring at room temperature for 30 min; then, it was transferred into an autoclave. The synthesis was repeated with 0.5 g copper acetate (Cu(Ac)_2_ 2H_2_O) as the precursor with 0.25 g of polyvinylpyrrolidone as the stabilizing agent. The further steps of the synthesis were the same as those described above.

The autoclave was heated to different temperatures (120 °C, 140 °C, 160 °C, and 180 °C, respectively) for 1 h and was kept for 6 h without shaking or stirring. After being cooled down to 25 °C (in 1 h), the precipitate was collected and washed with ethanol and Milli-Q water, and the products were dried at 40 °C overnight. The names of the samples are given in Table 1.

### 2.3. Calcination of CuxS Microparticles

The samples obtained at 180 °C were subjected to further heat treatment at 250 °C for 3 h (the heating and cooling periods were another 30 min respectively; the calculated average heating rate was 11.25 °C/min). To prevent the formation of copper oxides, the heat treatment was carried out in a Thermolyne F21100 Tube Furnace under Ar gas flow.

The abbreviation of the precursor salt appears in the superscript of the sample name (Ac in case of Cu(Ac)_2_, Cl in case of CuCl_2_), followed by the stabilizer agent (EDTA or PVP), and finally the C is labeled showing the heat treatment. The names of the samples are given in Table 1.

### 2.4. Characterization Methods

X-ray diffractograms (XRD) were recorded using a Rigaku Miniflex II diffractometer equipped (Prague, Czech Republic) with a Cu–Kα radiation source (λ = 1.5406 Å), and a graphite monochromator. Data points were taken in the 2θ° = 20–80° range at a scan speed of 1·(2θ°) min^−1^ [31]. The average mean primary crystallite size was calculated by using the Scherrer equation [32].

Scanning Electron Microscopy (SEM) micrographs were recorded with a Hitachi S-4700 Type II FE-SEM instrument (Tokyo, Japan), which operates using a cold field emission gun (5–15 kV). The size distribution of the particles was estimated from the SEM micrographs using the *ImageJ 1.52d* software (Bethesda, MD, USA).

Raman spectra were acquired by the Thermo Scientific DXR Raman microscope, (Tokyo, Japan) equipped with a diode-pumped frequency-doubled Nd:YAG laser with 10 mW maximum laser power. The sample was irradiated by laser with a wavelength of 532.2 nm.

N_2_ adsorption was used to calculate the specific surface areas of the samples, for which a BELCAT-A device (Osaka, Japan) was used to record the isotherms at 77 K via N_2_ adsorption.

### 2.5. Assessment of the Photocatalytic Efficiencies

A photoreactor system with 4 × 24 W visible light lamps (irradiation time = 2 h) was used to measure the photocatalytic activities. To exclude UV (λ < 400 nm) irradiation, the reactor was thermostated at 25 °C using 1 M NaNO_2_. This circulated solution absorbed UV photons below 400 nm, providing solely visible light irradiation. The photocatalyst suspension containing the pollutant (initial concentration of methyl orange (MO) C_0,MO_ = 30 µM; catalyst content C_photocatalyst_ = 1.0 g L^−1^; total volume of the suspension V_susp_ = 100 mL) was continuously purged with air to keep the concentration of dissolved oxygen constant during the whole experiment. The concentration decrease of the organic substrate was followed using an Agilent 8453 spectrophotometer (Agilent Technologies, Waldbronn, Germany) at 464 nm. The photocatalytic tests were repeated at least 2 times for each photocatalyst.

## 3. Results and Discussion

### 3.1. Structural Characterization of Cu_x_S Materials

**Figure 1:**
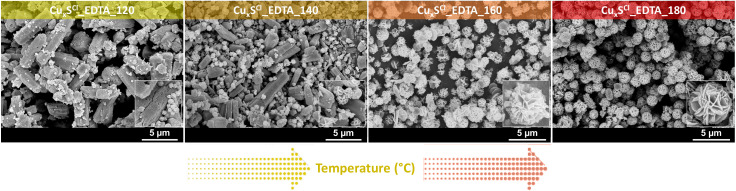
Effect of the increasing of the synthesis temperature (from 120 to 180 °C) on particle morphology and dispersity using CuCl_2_ as the precursor salt and EDTA as the stabilizing agent.

In the first step, the solvothermal synthesis temperature was determined by carrying out the crystallization procedure in the temperature range 120–180 °C. Based on the experiments, 180 °C was selected as a synthesis temperature, because at lower temperatures, the obtained particles were not isomorphic and neither monodisperse, as shown in Figure 1.

**Figure 2:**
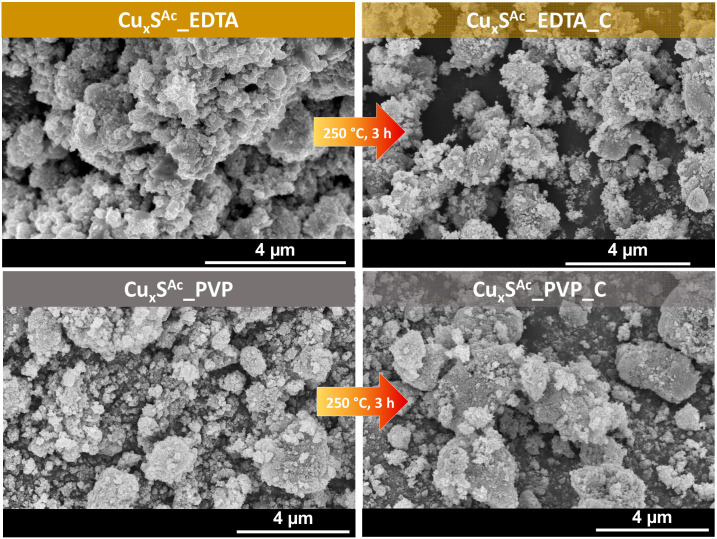
SEM micrographs of the obtained Cu_x_S samples prepared using Cu(OOCCH_3_)_2_·H_2_O as the precursor and different stabilizing agents (EDTA or PVP). The heat-treated samples (250 °C, 3 h) are also shown.

Consequently, Cu_x_S samples were prepared at 180 °C, and then their properties were explored. The morphology of the samples was investigated by scanning electron microscopy (SEM). Figure 2 shows SEM micrographs of microparticles obtained from Cu(Ac)_2_ in the presence of two different stabilizing agents (EDTA or PVP) [22,33] that influence the growth of the particles. There was no noticeable difference in the morphology; the samples consisted of micrometer-sized crystals (0.15 µm ± 0.3 µm), which were sometimes aggregated by changing the stabilizing agent. Moreover, after three hours of heat treatment, there were no significant geometric changes in the samples’ morphology (250 °C).

**Figure 3:**
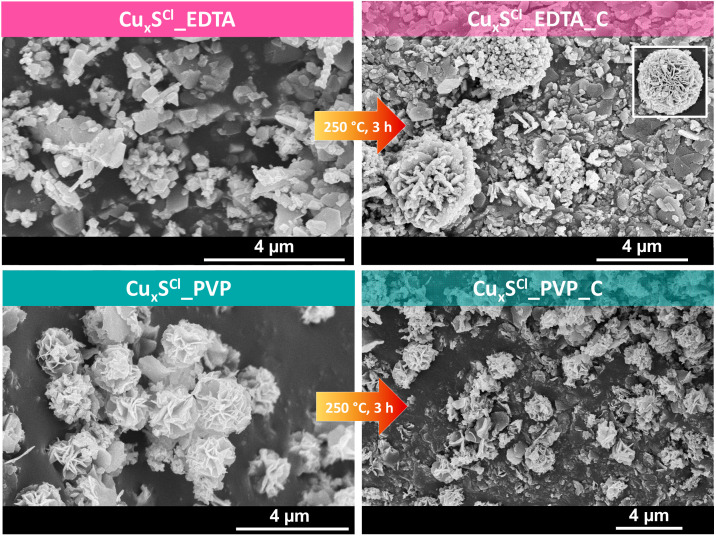
SEM micrographs of Cu_x_S samples prepared using CuCl_2_·2H_2_O as the precursor and different stabilizing agents (EDTA or PVP). The heat-treated samples (250 °C, 3h) are also shown.

Figure 3 shows the SEM micrographs of the microparticles obtained from CuCl_2_ with the same stabilizing agents (EDTA or PVP). The hierarchical structures were built from microplates, but among the samples, there were significant differences:

- with EDTA (Cu_x_S^Cl^_EDTA), the microparticles were plate-shaped, but after the heat treatment (Cu_x_S^Cl^_EDTA_C), microspheres made from plates appeared;
- using PVP as the stabilizing agent, the sample showed spherical morphology (Cu_x_S^Cl^_PVP), which was partially deteriorated after the heat treatment process (Cu_x_S^Cl^_PVP_C).

The formation mechanism of the Cu_x_S particles in the case of using PVP as a stabilizing agent is already known in the literature. Firstly, several crystallization nuclei are formed, which start to grow, allowing the formation of the nanosheets (their stabilization is assured by the presence of PVP). During the nanosheet formation, local defects may arise, which are suitable for the growth of other nanosheets in different directions, forming at the end Cu_2_S nanoflowers [34]. By using EDTA as a stabilizing agent, the mechanism of particle formation takes place in a different way. The process may include complexation of copper, which controls the nucleation speed. Moreover, the formed nanosheets are not stabilized by PVP; therefore, multiple branching was expected and observed [19,35].

**Figure 4:**
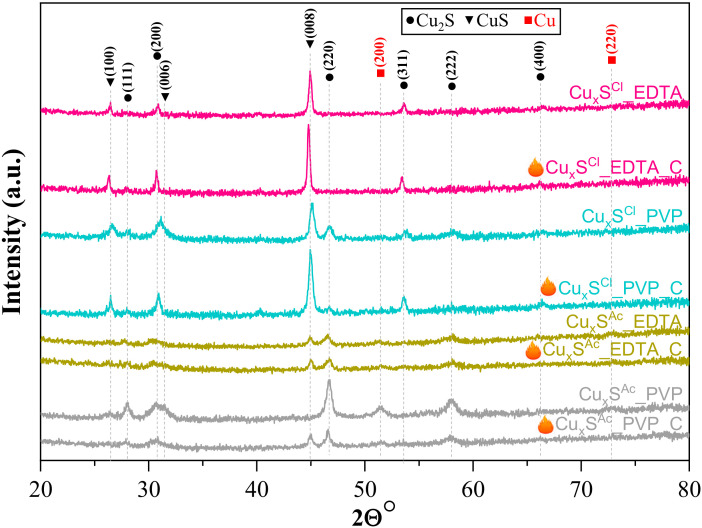
XRD patterns of all samples, showing the diffraction peaks of the materials (● Cu, ▼ CuS and ■ Cu_2_S) in the sample, indicating the results of XRD measurements after the heat treatment.

From the XRD patterns, it has been concluded that a composite system formed during the synthesis process. Figure 4 shows the XRD patterns of all the samples. (111), (200), (220), (311), (222), and (400) crystallographic planes of Cu_2_S (JCPDS card no. 84-1770) were identified alongside the diffraction peaks of CuS ((100), (006), and (008)) (JCPDS card no. 06-0464). Nevertheless, Cu was also detected and identified according to JCPDS card No. 85-1326 in case of Cu_x_S^Ac^_PVP.

From the XRD patterns, it was also calculated that the (100) crystallographic plane at 26 (2θ°) appeared when CuCl_2_·2H_2_O was used, and the values of the calculated areas were the following: *Cu_x_S^Cl^_EDTA < Cu_x_S^Cl^_EDTA_C < Cu_x_S^Cl^_PVP_C < Cu_x_S^Cl^_PVP*, which may be related to the rose-like shape that appeared in the scanning electron microscopic images.

The calculated average crystal size for the crystal side (008) for the base catalysts is 20–40 nm. From the values calculated by the Scherrer equation, after calcination, the size of the primary crystallite increases slightly (30–50 nm). This increase is systematically observed between the sample and the calcined counterpart, with an increase of approximately 10 nm per sample.

From the intensity of the diffraction peaks, it can be stated that the crystallinity of copper chloride-based samples can be considered higher. Interestingly, in these samples, the intensity of the peak responsible for the (008) crystallographic plane is more intense than in the samples obtained from copper–acetate.

**Figure 5:**
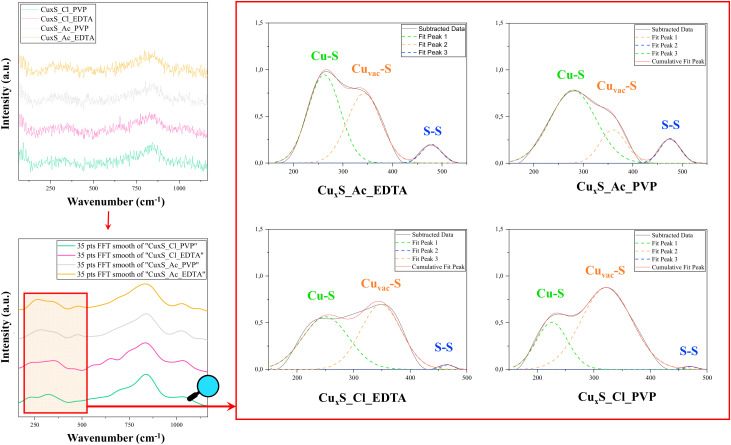
Raman spectra in the 150–1500 cm^−1^ region; the 35 pts Fast Fourier Transform FFT smoothed spectra of these samples in the 150–500 cm^−1^ region containing the Cu-S, Cu_vac_-S, and S-S vibration signals.

Therefore, Raman spectroscopy was applied to study the possible transformations of the crystalline structure in these compounds. Figure 5 shows the Raman spectra of the samples in 150–1500 cm^−1^ region, which revealed a peak at 476 cm^−1^, which can be assigned to vibrational (stretching) modes from the covalent S–S bonds. The intensity of this peak is higher for Cu(Ac)_2_ samples.

Another signal was identified at approximately 270 cm^−1^ that was attributed to the Cu–S bond vibration [36] and Cu vacancy neighboring sulfur Cu_2−x_S (0,6 ≤ x ≤ 1) phonon mode [37] at 322 cm^−1^, which is hereafter denoted as Cu_vac_S. It can be observed that the ratio of the intensities of the latter two peaks varies with the used precursor salt. The intensity of the peak characteristic of vacancies increases for the samples obtained from CuCl_2_. Since the appearance of vacancies can cause an improvement in photocatalytic performance [38,39,40], these observations can be positive projections in the evaluation of activity tests.

Furthermore, based on the classical vibrational frequency model of a diatomic molecule, the important shift of the Raman signal given by the vacancies presence into the sample Cu_x_S^Cl^_EDTA, in comparison with the corresponding Raman bands from 250 to 450 cm^−1^, can be associated with the existence of a high density of vacancies for this sample.

### 3.2. Investigation of Photocatalytic Properties of the Cu_x_S Materials

**Figure 6:**
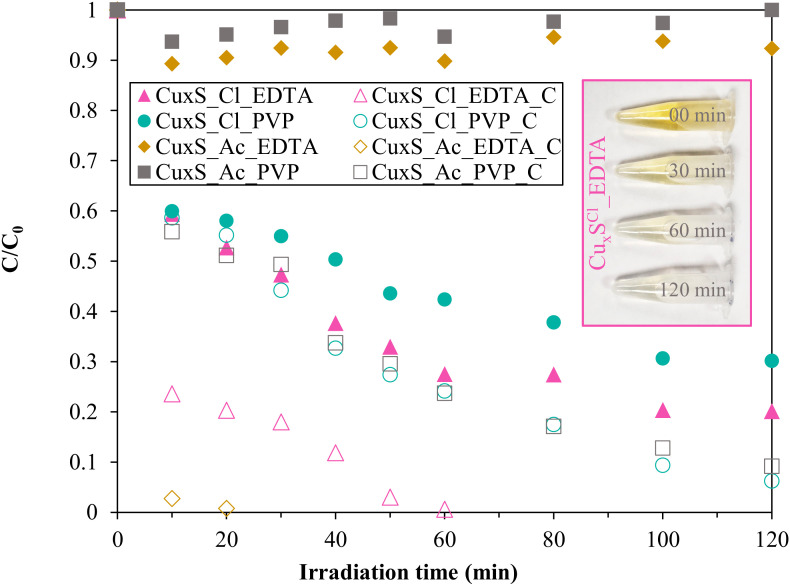
Photodegradation results with the Cu_x_S semiconductors under Vis light using methyl-orange as a model pollutant.

The efficiency of the Cu_x_S materials in the degradation of methyl orange showed (Figure 6) promising results, achieving apparently nearly 100% of conversion.

(1) The first approach was to compare materials synthesized from different precursors (without additional heat treatment): starting from copper acetate (Cu_x_S^Ac^_EDTA and Cu_x_S^Ac^_PVP), neither of the semiconductors were active. However, the samples prepared from copper (II) chloride (Cu_x_S^Cl^_EDTA and Cu_x_S^Cl^_PVP) showed significant activity toward the degradation of methyl orange, achieving 78% and 30% conversion values.
(2) As a result of the heat treatment, the activity of all the samples increased. Since the XRD and SEM results do not fully explain the differences in activity, the origin of this performance needed to be verified; therefore, the degree of adsorption was investigated, which might significantly affect the final degradation result. Therefore, it was essential to determine the specific surface area of all the samples.

As stated in the discussion of the Ramen results, the appearance of vacancies may also have an impact on activity. This observation was also confirmed for the basic catalysts, as the vacancy peaks for the Cu_x_S^Cl^_EDTA and Cu_x_S^Ac^_PVP samples were also higher compared to the other two samples, which is also consistent with the activity tests.

The possible photocatalytic mechanism in the case of Cu_x_S could be the hole (h^+^)-driven oxidation, due to the fact that the catalysts are showing remarkable adsorption properties toward methyl orange, making possible the direct contact between the surface of the catalyst and the organic molecule [41,42]. However, a radical-based mechanism should be not excluded totally, but it most probably is less dominant.

**Figure 7:**
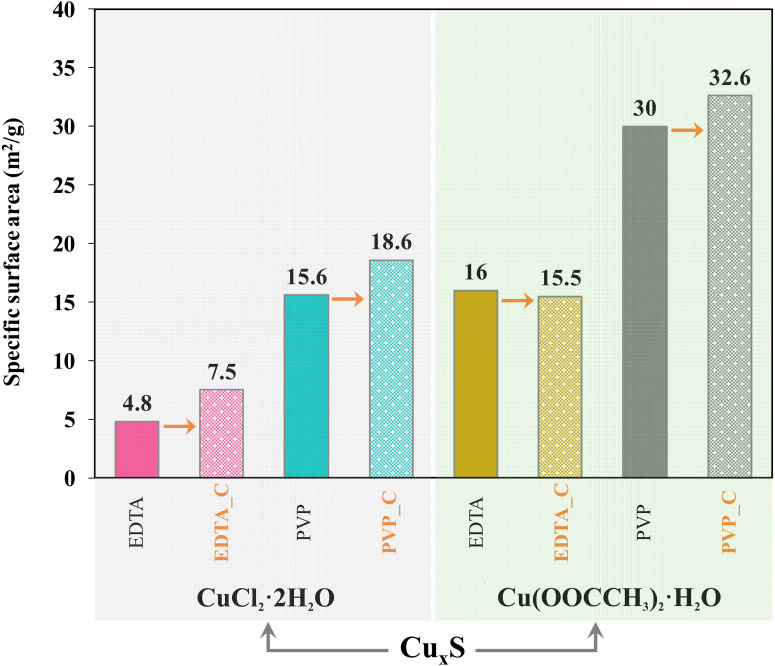
Brunauer–Emmett–Teller (BET) specific surface areas of the Cu_x_S samples.

The Brunauer–Emmett–Teller (BET) specific surface area values of Cu_x_S increased with the heat treatment, which was presumably because the hierarchical crystals deteriorated, and this phenomenon may affect the photocatalytic activity or the adsorption capacity of the samples. This phenomenon is the opposite of classical theory, since during heat treatment, the primary crystallite size usually increases, thereby decreasing the specific surface area. The obtained results (Figure 7) showed that although the surface area of the samples can increase the activity, this depends not only on this property. The change in specific surface area and the change in vacancies observed during Raman measurements are not a complete explanation for the conversion values, so the adsorption capacity of the samples should be examined as an additional measurement.

In the case of the calcined samples, the high conversion can also be caused by the higher adsorption (which can be linked to the specific surface area); thus, more detailed measurements were carried out to measure the contribution of the adsorption to the total MO removal.

Two samples with the highest conversion rates were selected (Cu_x_S^Cl^_EDTA_C and Cu_x_S^Ac^_EDTA_C) and compared through adsorption experiments with their untreated sample pairs: Cu_x_S^Cl^_EDTA and Cu_x_S^Ac^_EDTA. The adsorption tests were carried out at four different concentrations.

**Figure 8:**
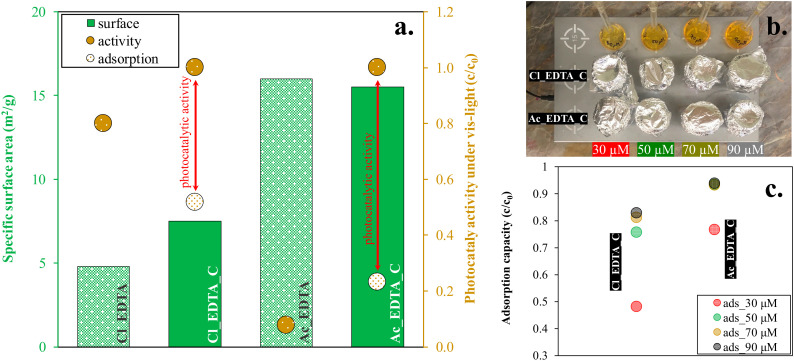
(**a**) The specific surface area (before and after the calcination step), the photocatalytic activity, and the measured adsorption results at 30 µM with the samples with the highest conversion value; (**b**) the adsorption capacity on four different concentration (30, 50, 70, and 90 µM), and (**c**) the measurement in the dark with constant stirring;

Figure 8 shows the increase in specific surface area, as well as the photocatalytic activity and the adsorption results for the selected samples. The figure illustrates that although the specific surface area may be related to the adsorption capacity of the samples, this phenomenon alone does not explain the increased conversion (the degree of concentration loss) value. The degree of photocatalytic activity can be seen (red interval), excluding the adsorption capacity. Thus, subtracting the adsorption from the activity yields the photocatalytic activity (red interval) of the sample.

After the photodegradation tests, the morphological and crystal structure properties of the used samples were measured to examine the stability of the samples. The XRD patterns showed also that the samples remained unchanged, although some minor differences in the diffraction peaks’ ratio was observed, suggesting a very slow photo-induced crystallization process. It can be observed that the catalysts retain not only their components but also their proportions after the test.

## 4. Conclusions

The subject of the present research was the synthesis of Cu_x_S samples, varying several synthesis procedures. The obtained morphology in the case of CuCl_2_ was microplate or spherical-like, while the heat treatment also contributed to the further development of the spherical morphology. In case of Cu(Ac)_2_, the particles were isomorphic—and by heat treatment, the aggregation of the particles was observed. Raman spectroscopy indicated that photocatalytic activity can be related to the Cu_vac_–S peak, so an increase in Cu vacancy promotes photocatalytic activity.

The materials also showed different crystallographic properties—the relative intensity of the peak responsible for the (008) crystallographic plane was more pronounced for samples with higher activity. Comparing the results obtained from the characterization methods and the photocatalytic activity values proved that the (008) crystallographic plane and the vacancies detected using Raman spectroscopy were responsible for the photocatalytic decomposition process.

## Figures and Tables

**Table 1:** The nomenclature, synthesis, and the heat treatment parameters of the Cu_x_S samples. EDTA: ethylenediaminetetraacetic acid, PVP: polyvinylpyrrolidone.

Sample	Precursor	Stabilizing Agent	Calcination
Cu_x_S^Ac^_EDTA	Cu(Ac)_2_·H_2_O	EDTA	ø
Cu_x_S^Ac^_EDTA_C			250 °C, 3 h
Cu_x_S^Ac^_PVP		PVP	ø
Cu_x_S^Ac^_PVP_C			250 °C, 3 h
Cu_x_S^Cl^_EDTA	CuCl_2_·H_2_O	EDTA	ø
Cu_x_S^Cl^_EDTA_C			250 °C, 3 h
Cu_x_S^Cl^_PVP		PVP	ø
Cu_x_S^Cl^_PVP_C			250 °C, 3 h
